# Targeting class I histone deacetylases by the novel small molecule inhibitor 4SC‐202 blocks oncogenic hedgehog‐GLI signaling and overcomes smoothened inhibitor resistance

**DOI:** 10.1002/ijc.31117

**Published:** 2017-11-06

**Authors:** Wolfgang Gruber, Elisabeth Peer, Dominik P. Elmer, Christina Sternberg, Suzana Tesanovic, Pedro del Burgo, Sonia Coni, Gianluca Canettieri, Daniel Neureiter, René Bartz, Hella Kohlhof, Daniel Vitt, Fritz Aberger

**Affiliations:** ^1^ Department of Molecular Biology Cancer Cluster Salzburg, Paris‐Lodron University of Salzburg Salzburg 5020 Austria; ^2^ Department of Molecular Medicine Sapienza University of Rome Rome 00161 Italy; ^3^ Institute of Pathology, Cancer Cluster Salzburg, Paracelsus Medical University, Salzburger Landeskliniken (SALK) Salzburg 5020 Austria; ^4^ 4SC AG Planegg‐Martinsried 82152 Germany; ^5^ Immunic AG Planegg‐Martinsried 82152 Germany

## Abstract

Aberrant activation of Hedgehog (HH)/GLI signaling is causally involved in numerous human malignancies, including basal cell carcinoma (BCC) and medulloblastoma. HH pathway antagonists targeting smoothened (SMO), an essential effector of canonical HH/GLI signaling, show significant clinical success in BCC patients and have recently been approved for the treatment of advanced and metastatic BCC. However, rapid and frequent development of drug resistance to SMO inhibitors (SMOi) together with severe side effects caused by prolonged SMOi treatment call for alternative treatment strategies targeting HH/GLI signaling downstream of SMO. In this study, we report that 4SC‐202, a novel clinically validated inhibitor of class I histone deacetylases (HDACs), efficiently blocks HH/GLI signaling. Notably, 4SC‐202 treatment abrogates GLI activation and HH target gene expression in both SMOi‐sensitive and ‐resistant cells. Mechanistically, we propose that the inhibition of HDACs 1/2/3 is crucial for targeting oncogenic HH/GLI signaling, and that class I HDAC inhibitors either in combination with SMOi or as second‐line therapy may improve the treatment options for HH‐associated malignancies with SMOi resistance.

## Abstract 2

**What's new?**

Inhibition of signaling by Hedgehog polypeptide and the effector protein GLI is an effective therapeutic strategy for basal cell carcinoma and medulloblastoma. The frequent development of resistance to Hedgehog pathway inhibitors (HPI), however, calls for the identification of alternative drugs. This study identifies the class I histone deacetylase inhibitor 4SC‐202 as a novel Hedgehog/GLI antagonist. A clinically suitable small molecule, 4SC‐202, showed inhibitory efficacy, blocking GLI activation and Hedgehog target gene expression, even in cells with acquired HPI resistance. The findings suggest that 4SC‐202 could be effective against Hedgehog‐driven cancers that are refractory to standard targeted therapy.

Hedgehog (HH)/GLI signaling plays a pivotal role in many human malignancies. Notably, targeted pathway inhibition has shown clinical success particularly in HH‐driven basal cell carcinoma (BCC) and medulloblastoma.^1^, ^2^ However, rapid and frequent development of resistance to HH inhibitors urgently calls for additional treatment options.^3^ Canonical HH/GLI signaling is initiated by the binding of secreted HH protein to Patched (PTCH), a transmembrane domain protein that represses HH signaling in its unliganded state by inhibiting the ciliary localization and activation of the essential HH effector Smoothened (SMO). Binding of HH to PTCH allows the translocation of SMO into the primary cilium, where activated SMO triggers the formation of active GLI transcription factors (GLI2 and GLI3) by preventing proteolytic GLI repressor formation and by releasing them from their repressor Suppressor of Fused (SUFU) (for reviews, see Refs.^4^, ^5^). Active full‐length GLI proteins translocate to the nucleus, where they induce HH target gene expression including the strong transcriptional activator GLI1 that not only amplifies HH signal strength but whose expression level also serves as robust readout for HH/GLI pathway activation.^6^

Active GLI proteins drive tumor formation and promote cancer progression by inducing proliferation, survival, self‐renewal and metastasis.^7^, ^8^ To target oncogenic HH/GLI signaling, small molecule SMO inhibitors (SMOi) have been developed. Vismodegib and sonidegib represent two FDA‐approved SMOi for the treatment of advanced and metastatic BCC, a nonmelanoma skin cancer driven by aberrant activation of HH/GLI signaling.^1^, ^9^ Despite striking therapeutic efficacy, severe side effects of SMOi drugs and frequent development of SMOi resistance pose major challenges to future HH pathway inhibitor therapies.^3^, ^10^, ^11^ Therefore, the identification of targets and drugs to be used in combination or as an alternative to SMOi, particularly in settings of SMOi‐resistance, is critical to improve anti‐HH‐therapies in oncology.

Targeting epigenetic regulators such as histone deacetylases (HDACs) has proven a successful therapeutic option in several malignancies. Intriguingly, HDACs have been shown to modulate the activity of HH/GLI signaling with evidence for both a positive and repressive impact, demanding selective inhibition of HDACs, which enhance oncogenic HH/GLI signaling.^12^, ^13^, ^14^, ^15^

In this study, we show that the clinically suitable class I HDAC inhibitor (HDACi) 4SC‐202 efficiently abrogates HH/GLI signaling in a human model of oncogenic HH/GLI signaling. Importantly, 4SC‐202 inhibited HH/GLI signaling in both, SMOi‐sensitive and SMOi‐resistant settings and interfered with the growth of HH/GLI‐dependent BCC cells *in vivo*, thereby identifying 4SC‐202 as a promising epigenetic drug for the treatment of HH‐driven cancers.

## Materials and Methods

For an extended version of Materials and Methods, see Supporting Information.

### Cell culture and *in vivo* assays

NIH/3T3 Gli reporter cells (AMS Biotechnology) were used to monitor Hh pathway activity in response to chemical treatments and cell viability was assayed in parallel.

To study human HH/GLI signaling, we applied Daoy medulloblastoma cells (ATCC HTB‐186) responsive to chemical and genetic pathway activation. The following chemicals were used: Smoothened agonist SAG (Axxora), vismodegib and entinostat (LC Laboratories), OG‐L002, SAHA/vorinostat and 4SC‐202 (4SC AG). Cell proliferation and viability were determined by Alamar Blue assays (see extended materials and methods).

*In vivo* experiments were done in NSG mice. Following randomization of mice with palpable tumors, mice were treated with 4SC‐202 (80 mg/kg/day per oral gavage) or solvent. Tumor volume was measured every 2–3 days using a caliper.

### RNA isolation and quantitative PCR (qPCR)

Total RNA was isolated using TRI reagent (Sigma‐Aldrich) according to manufacturer's protocol followed by LiCl precipitation. qPCR was done on a Rotor‐Gene Q (Qiagen) using GoTaq 2× qPCR Mastermix (Promega). qPCR primers are listed in Supporting Information, Table 1.

### Western blot analysis, chromatin immunoprecipitation (ChIP) and immunohistochemistry

For protein detection by Western blot analysis, the following primary antibodies were applied: anti‐GLI1 (V812), anti‐Beta Actin (D6A8), anti‐HDAC1 (D5C6U), anti‐HDAC2 (D6S5P), anti‐HDAC3 (7G6C5), anti‐p44/42 MAPK (Erk1/2), anti‐PCNA (D3H8P), anti‐Cyclin D1 (92G2), anti‐β‐Tubulin (9F3, all Cell Signaling) and anti‐SUFU (sc‐10933, Santa Cruz Biotechnology). ChIP assays were conducted using the SimpleChIP Kit (Cell Signaling) with cross‐linked chromatin immunoprecipitated overnight with either anti‐H3K27ac antibody (D5E4) or anti‐MYC‐tag antibody (9B11) or mouse IgG isotype control (Cell Signaling). Immunohistochemistry was done on FFPE tissue of three different skin specimens with diagnosis of BCC. Sections were stained using the following primary antibodies: anti‐HDAC1 (ab19845, Abcam 1:2000,), anti‐HDAC2 (ab16032, Abcam, 1:250) and anti‐HDAC3 (sc‐11417, Santa Cruz Biotechnology), 1:100).

### RNA interference

SUFU knockdown was done by lentiviral shRNA transduction as described in Ref. 16. The SUFU targeting shRNA construct was selected from the Mission TRC shRNA library (TRCN0000019466, Sigma) and a scrambled shRNA served as control (SHC002, Sigma). HDAC1, HDAC2 or HDAC3 and LSD1 expression was knocked down using the following ON‐TARGET plus siRNAs (siHDAC1 (L‐003493–00); siHDAC2 (L‐003495–02); siHDAC3 (L‐003496–00) and siLSD1 (KDMA1) (L‐009223–00–0005), Dharmacon), according to manufacturer's instructions. The ON‐TARGET plus nontargeting siRNA served as control (D‐001810–01–05, Dharmacon).

## Results

### 4SC‐202 inhibits canonical HH/GLI signaling in murine and human HH‐responsive cells

**Figure 1:**
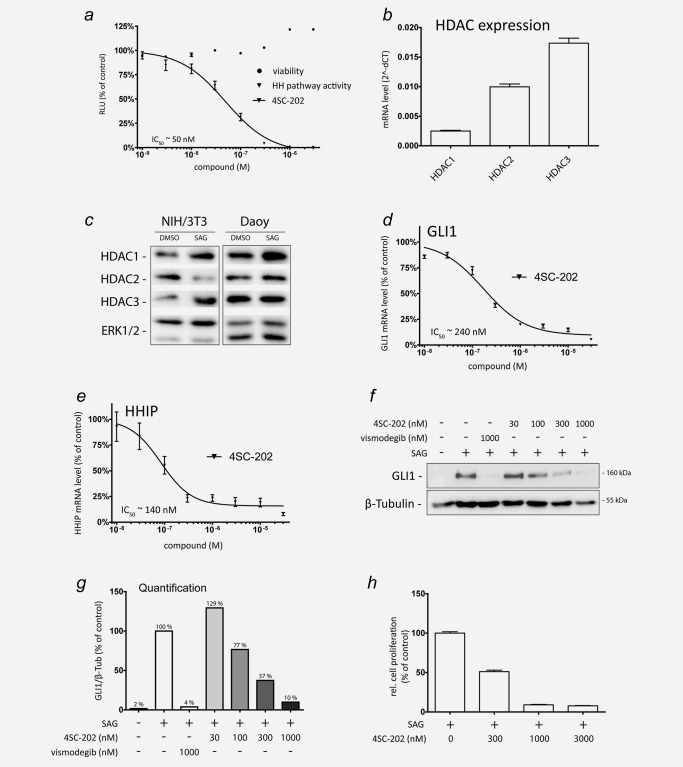
Inhibition of HH/GLI signaling by 4SC‐202. (*a*) Quantification of Hh/Gli signal strength in response to 4SC‐202 treatment using NIH/3T3 Hh reporter cells containing an 8x‐Gli binding site driving luciferase expression in response to Hh pathway activation. Hh/Gli luciferase reporter cells were stimulated with 1 μg/ml of recombinant Shh and treated with the respective 4SC‐202 concentrations. Luciferase activity and cell viability were measured after 24 hr of treatment. RLU: relative light units. (*b* and *c*) qPCR and Western blot analysis of HDAC1/2/3 mRNA levels in Daoy (*b*) and protein expression in HH‐responsive Daoy and NIH/3T3 cells (*c*). (*d* and *e*) qPCR analysis of GLI1 (*d*) and HHIP (*e*) mRNA expression in SAG‐stimulated Daoy cells exposed to increasing concentrations of 4SC‐202. GLI1 and HHIP mRNA levels in SAG‐treated Daoy cells without 4SC‐202 were set to 100%. (*f*) Western blot analysis of Daoy cells treated with respective concentrations of vismodegib or 4SC‐202. (*g*) Relative densitometric quantification of protein levels shown in (*f*). (*h*) Proliferation analysis of SAG‐stimulated Daoy cells in response to 4SC‐202 treatment at the concentrations indicated. β‐Tubulin or total ERK1/2 served as loading controls for Western blots.

4SC‐202 is a novel small‐molecule inhibitor selectively targeting class I HDACs 1/2/3 and the lysine‐specific demethylase LSD1.^17^ Notably, 4SC‐202 has shown excellent safety and promising anti‐leukemic activity in a recent phase I clinical trial (see TOPAS study at clinicaltrials.gov trial no. NCT01344707 and J Clin Oncol 32:5 sec, 2014 (suppl; abstr 8559)). To analyze whether 4SC‐202 affects HH/GLI signaling, we first treated Sonic Hh stimulated murine Hh/Gli luciferase reporter cells with 4SC‐202 and found that 4SC‐202 strongly repressed Gli activity with an IC_50_ of ∼50 nM (Fig. 1 *a*). To address the relevance of this finding to physiological HH/GLI signaling in a human context, we used HH responsive human medulloblastoma cells (i.e., Daoy) as a human model of canonical HH/GLI signaling in cancer cells.^18^ Daoy cells and NIH/3T3 express robust levels of HDAC1/2/3 (Figs. 1 *b* and 1 *c*) and activation of HH signaling by Smoothened agonist SAG induces HH target genes (e.g. GLI1 and HHIP) at the mRNA and protein level.^18^ Notably, 4SC‐202 treatment efficiently repressed SAG‐induced GLI1 (Fig. 1 *d*) and HHIP (Fig. 1 *e*) expression with an IC_50_ of ∼240 nM and ∼140 nM, respectively, without affecting primary cilia formation, an essential process for the coordination of HH/GLI signal activation (Supporting Information, Fig. 1).^19^ Analysis of GLI1 protein expression confirmed the concentration‐dependent repressive effect of 4SC‐202 on HH/GLI signaling (Figs. 1 *f* and 1 *g*). RNAi‐mediated inhibition of individual class I HDACs in Daoy cells suggests a dominant role for HDAC3, though the repressive effect on HH target gene activation was less pronounced when compared to 4SC‐202 treatment, most likely due to functional redundancy of class I HDACs (Supporting Information, Fig. 2). Furthermore, and in line with the proliferative role of HH/GLI in medulloblastoma, 4SC‐202 treatment also reduced the growth of SAG‐stimulated Daoy cells (Fig. 1 *h*).

### 4SC‐202 inhibits HH/GLI signaling by targeting class I HDACs

**Figure 2:**
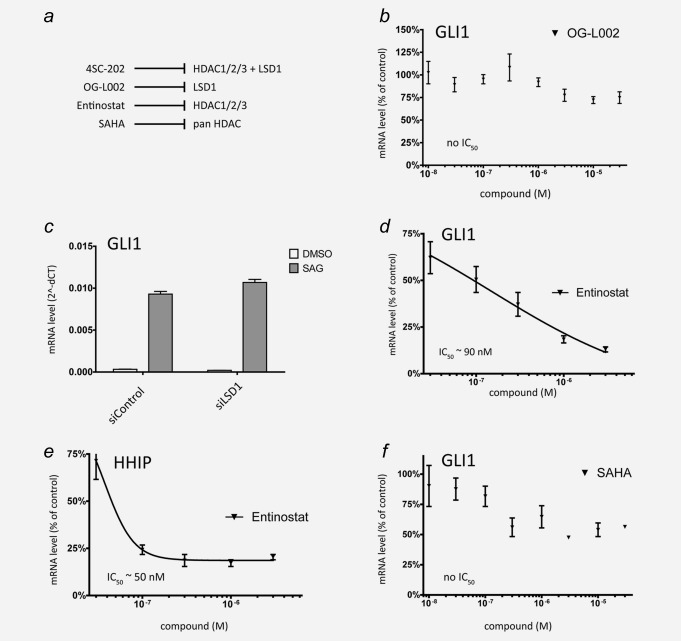
4SC‐202 inhibits HH/GLI signaling by targeting class I HDACs rather than LSD1. (*a*) Summary of inhibitors used for chemical perturbations and their molecular targets. (*b*) qPCR analysis of GLI1 mRNA expression in SAG‐stimulated Daoy cells in response to increasing concentrations of the LSD1 blocker OG‐L002. (*c*) GLI1 mRNA expression analysis in SAG‐induced or control‐treated Daoy cells transfected with control siRNA (siControl) or siRNA against LSD1 (siLSD1). (*d*, *e*) qPCR analysis of GLI1 (*d*) and HHIP (*e*) mRNA expression in SAG‐stimulated Daoy cells in response to the HDAC1/2/3 inhibitor entinostat. (*f*) GLI1 mRNA expression in Daoy cells treated with the FDA‐approved pan‐HDAC inhibitor SAHA (vorinostat).

4SC‐202 displays a unique inhibitor profile blocking not only the class I HDACs HDAC1/2/3, but also the lysine‐specific demethylase LSD1 (Fig. 2 *a*), which has been shown to play key roles in many malignancies. To investigate whether 4SC‐202 represses HH/GLI by inhibiting LSD1, we first tested the potent LSD1 inhibitor OG‐L002^20^ on SAG‐stimulated Daoy cells. As shown in Figure 2 *b*, OG‐L002 only moderately repressed SAG‐induced HH signaling to 75% of controls as monitored by GLI1 mRNA expression analysis. Furthermore, siRNA‐mediated knockdown of LSD1 (Supporting Information, Fig. 3) did not reduce GLI1 mRNA levels in response to SAG stimulation (Fig. 2 *c*). Thus, we conclude that the repressive effect of 4SC‐202 on HH/GLI signaling does not involve inhibition of LSD1. To further support the requirement of class I HDACs in HH/GLI signaling, we treated SAG‐stimulated Daoy cells with the selective HDAC1/2/3 inhibitor entinostat.^21^ Similar to 4SC‐202, entinostat effectively reduced GLI1 and HHIP expression in a concentration‐dependent manner (Figs. 2 *d* and 2 *e*). By contrast, treatment with the FDA‐approved pan‐HDAC inhibitor SAHA (vorinostat) was unable to efficiently abrogate HH/GLI signaling (Fig. 2 *f*), which may be a consequence of targeting HDAC6, whose inhibition has been shown to promote ciliogenesis and HH signaling.^14^ Together, we conclude that 4SC‐202 represses HH/GLI signaling by blocking class I HDACs, in line with a recent report identifying HDAC1/2 as important positive effectors of HH/GLI signaling in a murine model of medulloblastoma.^15^

### 4SC‐202 represses HH/GLI signaling in SMO‐inhibitor‐resistant human cancer cells

**Figure 3:**
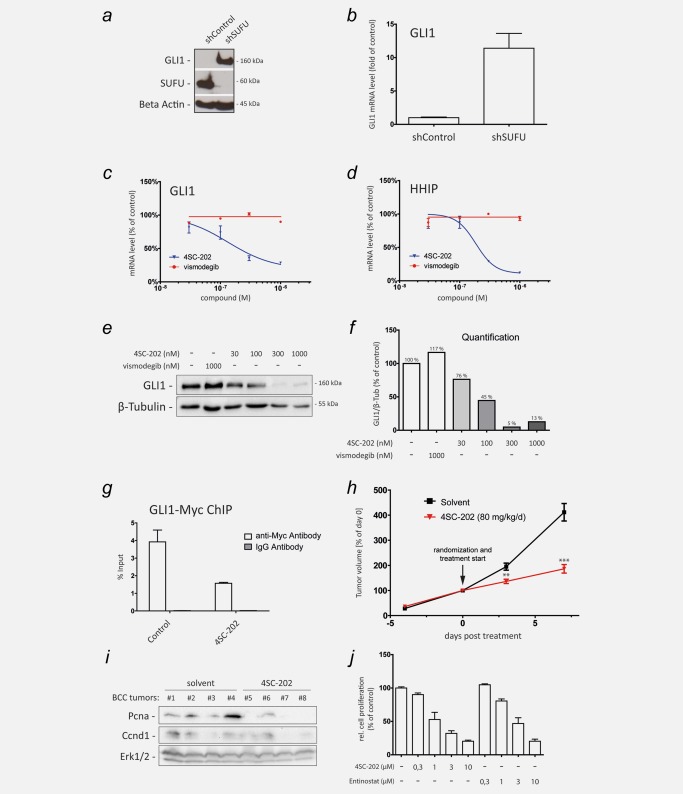
4SC‐202 inhibits HH/GLI signaling in SMOi‐resistant cancer cells. (*a*) Western blot analysis of GLI1 expression in cells with lentiviral shRNA‐mediated knockdown of SUFU (shSUFU) or control knockdown (shControl). Beta Actin served as loading control. (*b*) qPCR analysis of GLI1 mRNA expression in SUFU knockdown (shSUFU) and control cells (shControl) transduced with lentiviral nontargeting scrambled shRNA. (*c* and *d*) qPCR analysis of GLI1 (*c*) and HHIP mRNA expression (*d*) as readout for HH/GLI signaling activity in SUFU‐depleted SMOi‐resistant cells showing resistance to vismodegib but sensitivity to 4SC‐202 treatment. (*e*) Western blot analysis of SUFU‐depleted Daoy cells treated with vismodegib or 4SC‐202 at the concentrations indicated. β‐Tubulin served as loading control. Vismodegib was unable to reduce GLI1 protein levels, while 4SC‐202 treatment effectively abolished GLI1 protein expression. (*f*) Relative densitometric quantification of GLI1/β‐Tubulin protein levels shown in (*e*). (*g*) ChIP analysis of MYC‐tagged GLI1 binding to the GLI target promoter PTCH in response to control or 4SC‐202 treatment. Enrichment of GLI1 bound promoter DNA was measured by qPCR. IgG isotype antibody was used as control. (*h*) Murine BCC cells were subcutaneously injected into dorsal flanks of 12 NSG mice. 4SC‐202 was administered by oral gavage at 80 mg/kg/day. The tumor volume at day 0 (i.e., start of drug treatment (arrow)) was set to 100%. (*i*) Western blot analysis of solvent (allografts #1–#4) and 4SC‐202 treated (allografts #5–#8) BCC lysates probed for proliferation‐cell‐nuclear‐antigen (Pcna) and Ccnd1 expression. Erk1/2 expression served as loading control. (*j*) Analysis of *in vitro* cell proliferation of murine BCC cells in response to 4SC‐202 and entinostat treatment. ChIP: chromatin immunoprecipitation. **p < 0.01, ***p < 0.001.

HH/GLI pathway inhibitors targeting SMO (SMOi) have been approved for the treatment of advanced and metastatic BCC.^22^ However, frequent development of resistance to SMOi urgently calls for further drug targets and drugs blocking HH/GLI signaling downstream and independent of SMO activity and resistance status.^11^ HDACs have previously been shown to activate GLI proteins by modifying their acetylation status.^12^, ^15^ We thus hypothesized that 4SC‐202 interferes with oncogenic HH/GLI signaling downstream of SMO at the level of the GLI transcription factors that would allow HH/GLI targeting even in SMOi resistant cells. To this end, we first generated SMOi‐resistant Daoy cells by genetically depleting the endogenous GLI inhibitor SUFU. As shown in Figures 3 *a* and 3 *b*, SUFU‐depleted Daoy cells express high levels of GLI1 protein and mRNA independent of SAG or SHH stimulation. SUFU‐deficient Daoy cells were resistant to the FDA‐approved SMOi vismodegib, as evidenced by the inability of vismodegib to reduce the direct HH targets GLI1 and HHIP (Figs. 3 *c* and 3 *d*). Notably and in contrast to vismodegib, 4SC‐202 treatment reduced the expression of GLI1 and HHIP also in SMOi‐resistant Daoy cells at IC_50_ concentrations similar to those determined in the SMO‐dependent wild‐type Daoy model (Figs. 3 *c* and 3 *d*, compared to Figs. 1 *d* and 1 *e*). We confirmed these observations also at the protein level by showing that 4SC‐202, but not vismodegib, was able to reduce GLI1 protein expression in SUFU‐deficient Daoy cells (Figs. 3 *e* and 3 *f*). Consistently, 4SC‐202 also repressed the growth of both SMOi sensitive (wild‐type) and SMOi‐resistant (shSUFU) Daoy cells in a concentration‐dependent manner (Supporting Information, Fig. 4).

Acetylation of GLI1 has been suggested to decrease its DNA binding capacity.^12^ We therefore applied chromatin immunoprecipitation (ChIP) of GLI1 to test whether HDAC1/2/3 inhibition by 4SC‐202 is able to reduce GLI1 binding to the promoter of the GLI1 target PTCH. We found that 4SC‐202 treatment markedly reduced binding of GLI1 to the PTCH target promoter (Fig. 3 *g*), despite enhancing transcriptional activation marks (i.e., histone 3‐K27 acetylation) (Supporting Information, Fig. 5). These data corroborate the findings by Canettieri *et al*. (2010)^12^ and are in line with the hypothesis that HDAC inhibition by 4SC‐202 decreases the DNA‐binding capacity of GLI1 by increasing its acetylation, although we failed to reliably detect changes in endogenous GLI acetylation due to the low GLI expression levels in Daoy cells.

To analyze a possible effect of 4SC‐202 treatment on GLI processing, we used Ptch1 deficient Med1 cells derived from a genetic mouse model of medulloblastoma. Notably, treatment of Med1 cells with 4SC‐202 not only reduced Gli1 expression, but also reduced the ratio of Gli3 full‐length activator (Gli3A) to Gli3 repressor (Gli3R), suggesting that acetylation may also contribute to efficient GLI repressor formation in addition to its effect on DNA binding of GLI transcription factors (Supporting Information, Figs. 6*a* and 6*b*).

Having shown a pronounced inhibitory effect of 4SC‐202 on HH/GLI signaling and proliferation downstream of SMO, we next investigated whether 4SC‐202 treatment can repress the *in vivo* growth of HH/GLI dependent BCC cells in a preclinical *in vivo* model. To this end, we subcutaneously injected into immunodeficient NOD‐scid IL2Rgamma^null^ (NSG) mice *ptch1/p53* double‐deficient murine BCC cells expressing readily detectable levels of Hdac1/2/3 in a Hh/Gli independent manner as assessed by vismodegib treatment (Supporting Information, Fig. 7). When tumors reached an average volume of 400 mm^3^, we randomized the mice into two groups. The verum group received 80 mg/kg/day of 4SC‐202 in 2% methocel solution, the control group was treated with solvent only. Notably, treatment of BCC bearing mice with 4SC‐202 significantly decelerated *in vivo* tumor growth (Fig. 3 *h*), and markedly repressed the expression of the proliferation marker PCNA, which was paralleled by a moderate reduction of the Hh/Gli target gene and cell cycle regulator Cyclin d1 (Ccnd1)^7^ (Fig. 3 *i*). To support a direct effect of 4SC‐202 on the growth of murine BCC cells, we treated *ptch1/p53*‐deficient cells also *in vitro* and found reduced cellular proliferation in response to 4SC‐202 as well as entinostat (Fig. 3 *j*).

Together with high level expression of HDAC1 and HDAC2 in human BCC samples (Supporting Information, Fig. 8), our findings therefore warrant further studies of 4SC‐202 and other class I HDACi as combination or second‐line drugs to tackle the problem of frequent SMOi‐resistance development observed in HH‐driven cancers such as BCC.

## Discussion

SMOi provide clear clinical benefit to BCC patients, though rapid development of SMOi resistance and *a priori* insensitivity to SMO antagonists limit the therapeutic efficacy of SMO targeting.^11^ The lack of efficacy can, at least in part, be attributed to the many possibilities of malignant cells to activate GLI transcription factors in a noncanonical (SMO‐independent) manner. Examples for potent noncanonical and SMO‐independent GLI activator signals include TGFβ, RAS, MAPK/ERK, DYRKs, PI3K/AKT, EWS‐FLI1 and others.^5^, ^23^

In this study, we identify the potent and clinically suitable class I HDACi, 4SC‐202, as novel inhibitor of HH/GLI signaling in human cancer cells. Importantly, 4SC‐202 but not the FDA‐approved inhibitor vismodegib, efficiently abrogated HH/GLI signaling in SUFU‐depleted human medulloblastoma cells resistant to SMOi‐therapy. The SMO‐independent mode of action of 4SC‐202 may lead to benefits for patients, who do not qualify for therapy with SMOi either due to acquired or *a priori* SMOi resistance. Inhibition of GLI transcription factors is also an attractive strategy as GLI transcription factors frequently integrate with other oncogenic signaling cascades, thereby contributing to cancer progression and malignant traits of cancer and cancer stem cells.^5^

Mechanistically, our results are in agreement with a previous study by Canettieri *et al*., who showed that acetylation of GLI proteins negatively regulates their DNA‐binding capacity.^24^ In fact, we found that 4SC‐202 treatment strongly reduced binding of the GLI1 oncogene to the PTCH target promoter despite inducing an active chromatin as evidenced by increased H3K27 acetylation levels. In addition, class I HDACs may also act at the level of GLI processing, as we found that 4SC‐202 treatment increased the level of Gli3R in Ptch1‐deficient mouse medulloblastoma cells. Whether this is due to increased Gli3 acetylation remains to be addressed in follow‐up studies.

Previous studies have also identified interactions between different HDACs and HH/GLI signaling, though with partially different results. Canettieri *et al*. demonstrated a positive feedforward loop involving upregulation of HDAC1 expression in response to HH/GLI,^12^ while Dhanyamraju *et al*. describe a positive role of HDAC6 in the regulation of the mammalian HH pathway.^13^ By contrast, inhibition of HDAC6 has also been shown to restore primary ciliogenesis, thereby compensating for reduced HH signaling in STK11/LKB1‐depleted cells.^14^ HDAC6 may therefore exert context‐dependent positive and negative functions in the regulation of the HH/GLI pathway, which may also explain the failure of the FDA‐approved pan‐HDACi SAHA/vorinostat to efficiently inhibit HH/GLI signaling in medulloblastoma cells.

Intriguingly, analysis of the genetic landscape of SHH subgroup medulloblastoma identified frequent somatic alterations in genes encoding histone acetyl transferases.^25^ In light of these findings, our data together with the previous studies by the Canettieri group may provide a mechanistic basis for this observation and guide possible new therapeutic approaches.

The clinical relevance of our findings is further underlined by a recent phase I clinical trial with 4SC‐202, where 18 out of 24 patients with advanced hematologic malignancies went into follow up treatment, one patient showed complete response and remained on study medication for >28 months, while another patient had a partial response over a period of 8 months (Clinical trial: NCT01344707, poster J Clin Oncol 32:5 sec, 2014 (suppl; abstr 8559)). Although it is unclear whether the promising clinical response to 4SC‐202 treatment was due to inhibition of GLI activity, the results of the present study warrant further clinical evaluation of 4SC‐202 as a treatment for GLI‐dependent cancers including BCC, medulloblastoma and possibly other malignancies with high medical need.

## Author Contributions

WG, EP, DPE, CS, ST, PDB, DN, SC, GC and HK performed experiments. WG, RB, HK, DV, GC and FA designed experiments and analyzed data. WG and FA wrote the article. All authors critically reviewed the article before submission.

## Supporting information

Supporting Information

## References

[1] Von Hoff DD , LoRusso PM , Rudin CM , et al. Inhibition of the hedgehog pathway in advanced basal‐cell carcinoma. N Engl J Med 2009;361:1164–72. 19726763 10.1056/NEJMoa0905360

[2] Rudin CM , Hann CL , Laterra J , et al. Treatment of medulloblastoma with hedgehog pathway inhibitor GDC‐0449. N Engl J Med 2009;361:1173–8. 19726761 10.1056/NEJMoa0902903 PMC5317279

[3] Basset‐Seguin N , Sharpe HJ , de Sauvage FJ. Efficacy of Hedgehog pathway inhibitors in basal cell carcinoma. Mol Cancer Ther 2015;14:633–41. 25585509 10.1158/1535-7163.MCT-14-0703

[4] Mukhopadhyay S , Rohatgi R. G‐protein‐coupled receptors, Hedgehog signaling and primary cilia. Semin Cell Dev Biol 2014;33:63–72. 24845016 10.1016/j.semcdb.2014.05.002 PMC4130902

[5] Aberger F , Ruiz IAA. Context‐dependent signal integration by the GLI code: the oncogenic load, pathways, modifiers and implications for cancer therapy. Semin Cell Dev Biol 2014;33:93–104. 24852887 10.1016/j.semcdb.2014.05.003 PMC4151135

[6] Teglund S , Toftgard R. Hedgehog beyond medulloblastoma and basal cell carcinoma. Biochim Biophys Acta 2010;1805:181–208. 20085802 10.1016/j.bbcan.2010.01.003

[7] Kasper M , Regl G , Frischauf AM , et al. GLI transcription factors: mediators of oncogenic Hedgehog signalling. Eur J Cancer 2006;42:437–45. 16406505 10.1016/j.ejca.2005.08.039

[8] Clement V , Sanchez P , de Tribolet N , et al. HEDGEHOG‐GLI1 signaling regulates human glioma growth, cancer stem cell self‐renewal, and tumorigenicity. Curr Biol 2007;17:165–72. 17196391 10.1016/j.cub.2006.11.033 PMC1855204

[9] Sekulic A , Migden MR , Oro AE , et al. Efficacy and safety of vismodegib in advanced basal‐cell carcinoma. N Engl J Med 2012;366:2171–9. 22670903 10.1056/NEJMoa1113713 PMC5278761

[10] Tang JY , Mackay‐Wiggan JM , Aszterbaum M , et al. Inhibiting the hedgehog pathway in patients with the basal‐cell nevus syndrome. N Engl J Med 2012;366:2180–8. 22670904 10.1056/NEJMoa1113538 PMC4362529

[11] Atwood SX , Sarin KY , Whitson RJ , et al. Smoothened variants explain the majority of drug resistance in basal cell carcinoma. Cancer Cell 2015;27:342–53. 25759020 10.1016/j.ccell.2015.02.002 PMC4357167

[12] Canettieri G , Di Marcotullio L , Greco A , et al. Histone deacetylase and Cullin3‐REN(KCTD11) ubiquitin ligase interplay regulates Hedgehog signalling through Gli acetylation. Nat Cell Biol 2010;12:132–42. 20081843 10.1038/ncb2013

[13] Dhanyamraju PK , Holz PS , Finkernagel F , et al. Histone deacetylase 6 represents a novel drug target in the oncogenic Hedgehog signaling pathway. Mol Cancer Ther 2015;14:727–39. 25552369 10.1158/1535-7163.MCT-14-0481

[14] Jacob LS , Wu X , Dodge ME , et al. Genome‐wide RNAi screen reveals disease‐associated genes that are common to Hedgehog and Wnt signaling. Sci Signal 2011;4:ra4. 21266715 10.1126/scisignal.2001225 PMC3790583

[15] Coni S , Mancuso AB , Di Magno L , et al. Selective targeting of HDAC1/2 elicits anticancer effects through Gli1 acetylation in preclinical models of SHH Medulloblastoma. Sci Rep 2017;7:44079. 28276480 10.1038/srep44079 PMC5343431

[16] Kasper M , Regl G , Eichberger T , et al. Efficient manipulation of Hedgehog/GLI signaling using retroviral expression systems. Methods Mol Biol 2007;397:67–78. 18025714 10.1007/978-1-59745-516-9_6

[17] Pinkerneil M , Hoffmann MJ , Kohlhof H , et al. Evaluation of the therapeutic potential of the novel isotype specific HDAC inhibitor 4SC‐202 in urothelial carcinoma cell lines. Target Oncol 2016;11:783–98. 27250763 10.1007/s11523-016-0444-7 PMC5153417

[18] Gotschel F , Berg D , Gruber W , et al. Synergism between Hedgehog‐GLI and EGFR signaling in Hedgehog‐responsive human medulloblastoma cells induces downregulation of canonical Hedgehog‐target genes and stabilized expression of GLI1. PLoS One 2013;8:e65403. 23762360 10.1371/journal.pone.0065403 PMC3677915

[19] Eggenschwiler JT , Anderson KV. Cilia and developmental signaling. Annu Rev Cell Dev Biol 2007;23:345–73. 17506691 10.1146/annurev.cellbio.23.090506.123249 PMC2094042

[20] Liang Y , Quenelle D , Vogel JL , et al. A novel selective LSD1/KDM1A inhibitor epigenetically blocks herpes simplex virus lytic replication and reactivation from latency. MBio 2013;4:e00558–12. 23386436 10.1128/mBio.00558-12 PMC3565832

[21] Huang PH , Chen CH , Chou CC , et al. Histone deacetylase inhibitors stimulate histone H3 lysine 4 methylation in part via transcriptional repression of histone H3 lysine 4 demethylases. Mol Pharmacol 2011;79:197–206. 20959362 10.1124/mol.110.067702 PMC3014276

[22] Dlugosz A , Agrawal S , Kirkpatrick P. Vismodegib. Nat Rev Drug Discov 2012;11:437–8. 22653209 10.1038/nrd3753 PMC3383648

[23] Gruber W , Hutzinger M , Elmer DP , et al. DYRK1B as therapeutic target in Hedgehog/GLI‐dependent cancer cells with Smoothened inhibitor resistance. Oncotarget 2016;7:7134–48. 26784250 10.18632/oncotarget.6910 PMC4872774

[24] Coni S , Antonucci L , D'amico DD , et al. Gli2 acetylation at lysine 757 regulates hedgehog‐dependent transcriptional output by preventing its promoter occupancy. PLoS One 2013;8:e65718. 23762415 10.1371/journal.pone.0065718 PMC3675076

[25] Northcott PA , Buchhalter I , Morrissy AS , et al. The whole‐genome landscape of medulloblastoma subtypes. Nature 2017;547:311–7. 28726821 10.1038/nature22973 PMC5905700

