# Supplementary material for: Targeting class I histone deacetylases by the novel small molecule inhibitor 4SC‐202 blocks oncogenic hedgehog‐GLI signaling and overcomes smoothened inhibitor resistance
Source: Int J Cancer. 2017 Nov 6;142(5):968–75. doi: 10.1002/ijc.31117 (PMC5813224; doi:10.1002/ijc.31117)
Supplement: Supplementary file 1 — Supporting Information [file IJC-142-968-s001.pdf]

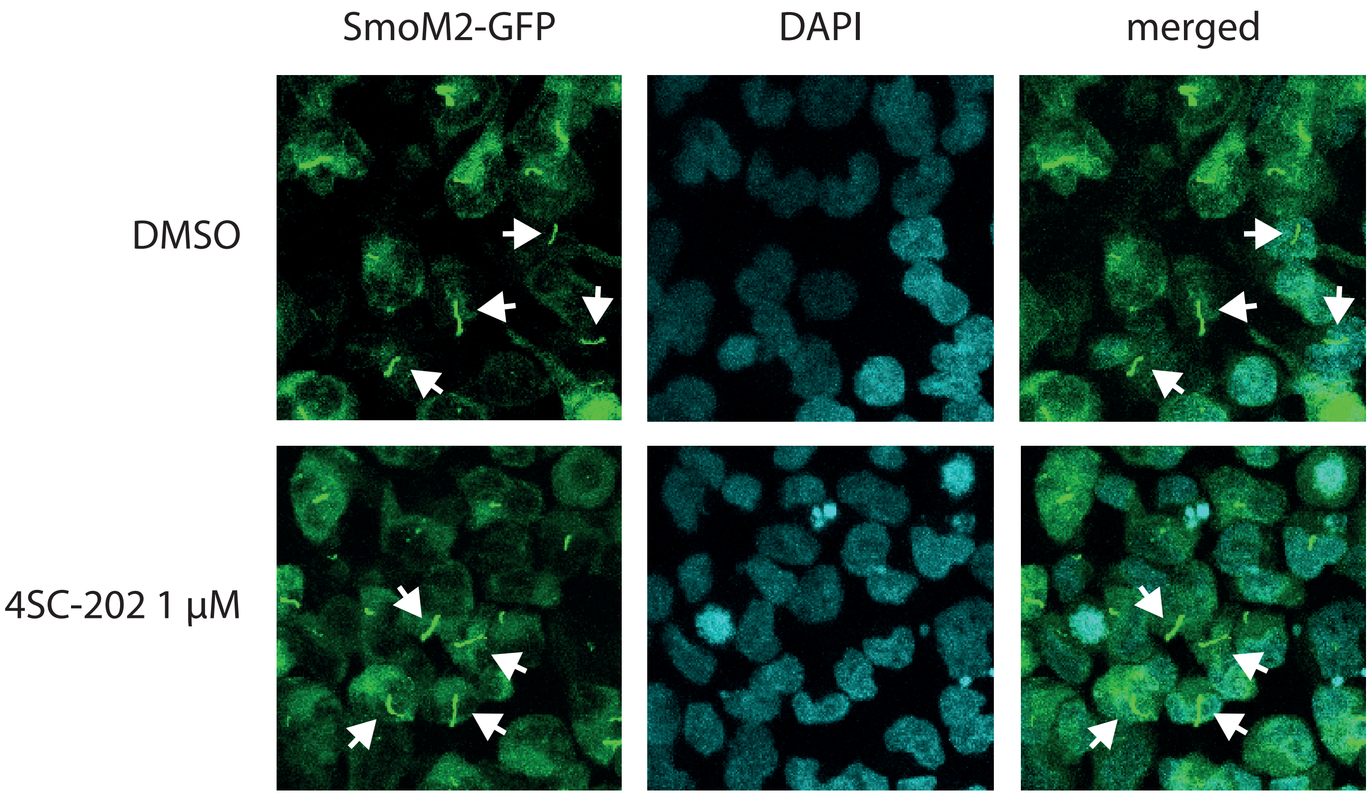

Gruber et al., suppl Figure 1

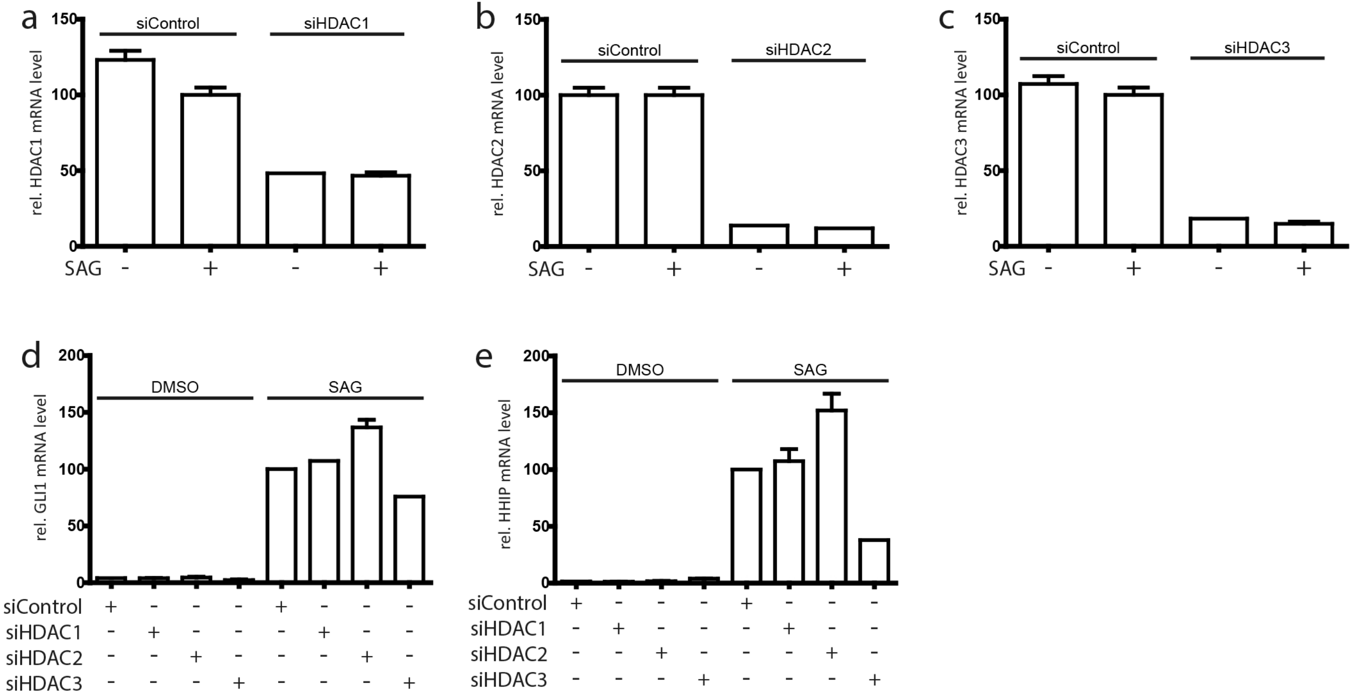

Gruber et al., suppl Figure 2

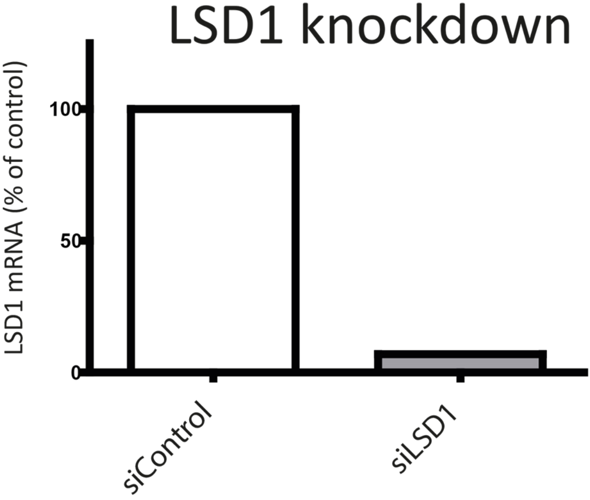

Gruber et al., suppl Figure 3

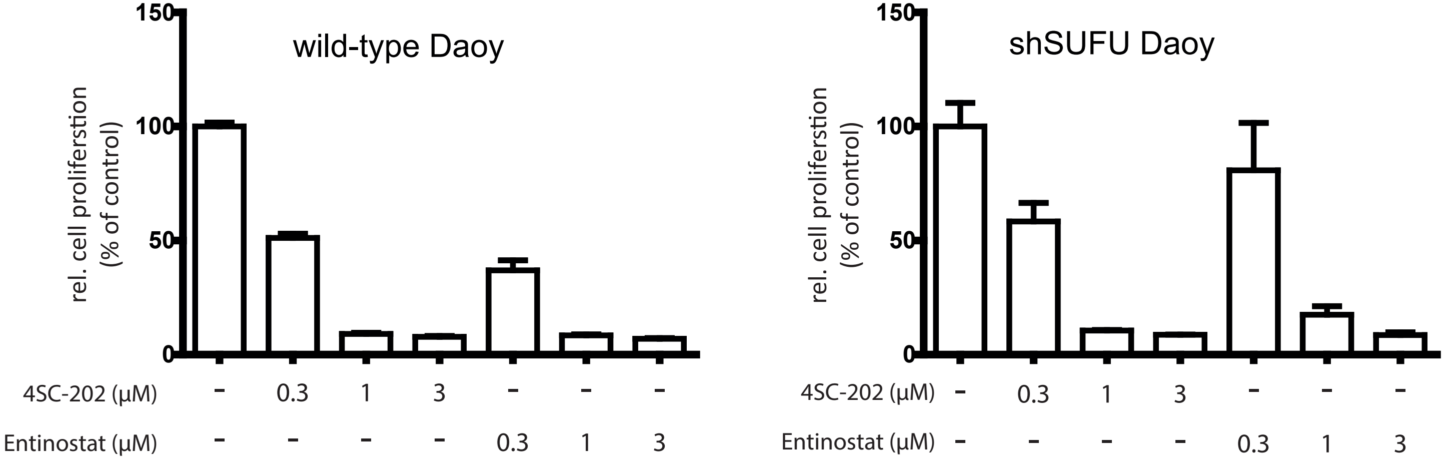

Gruber et al., suppl Figure 4

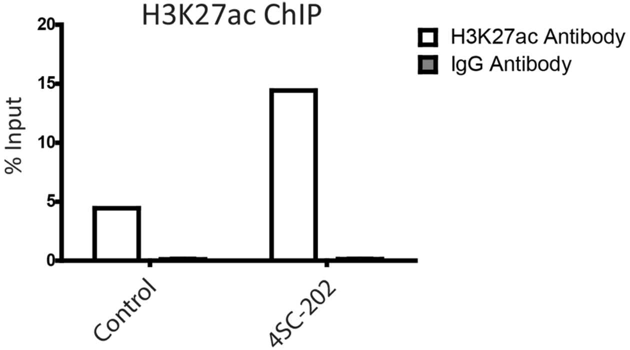

Gruber et al., Suppl. Figure 5

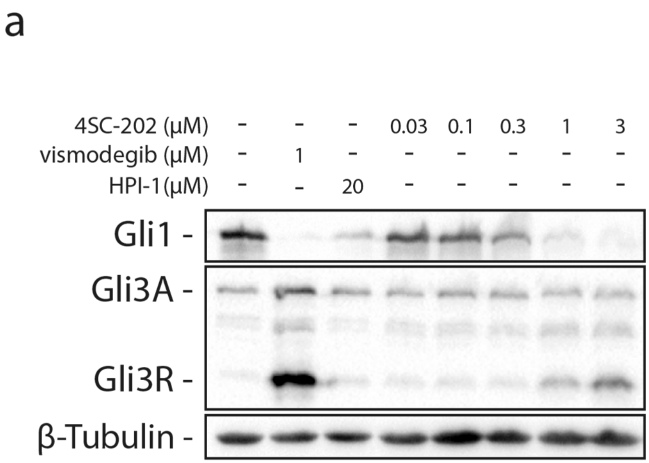

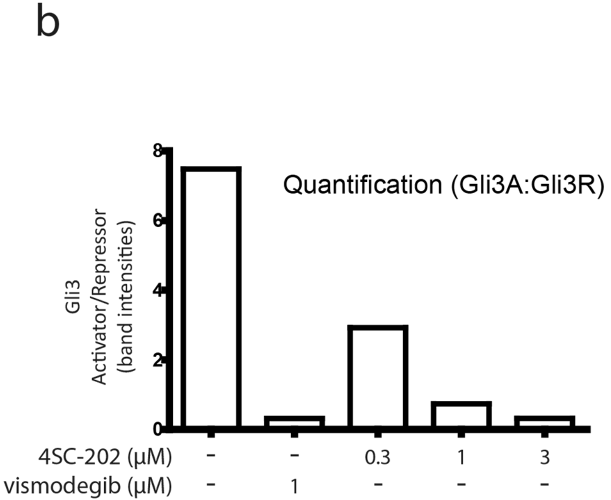

Gruber et al., Suppl. Figure 6

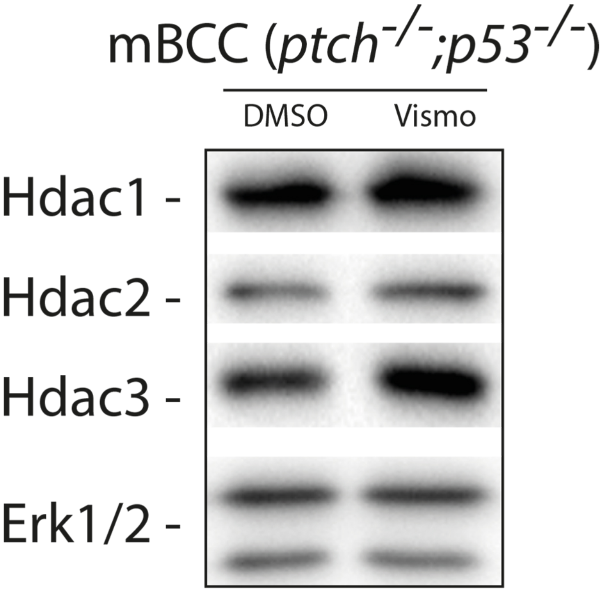

Gruber et al., suppl. Figure 7

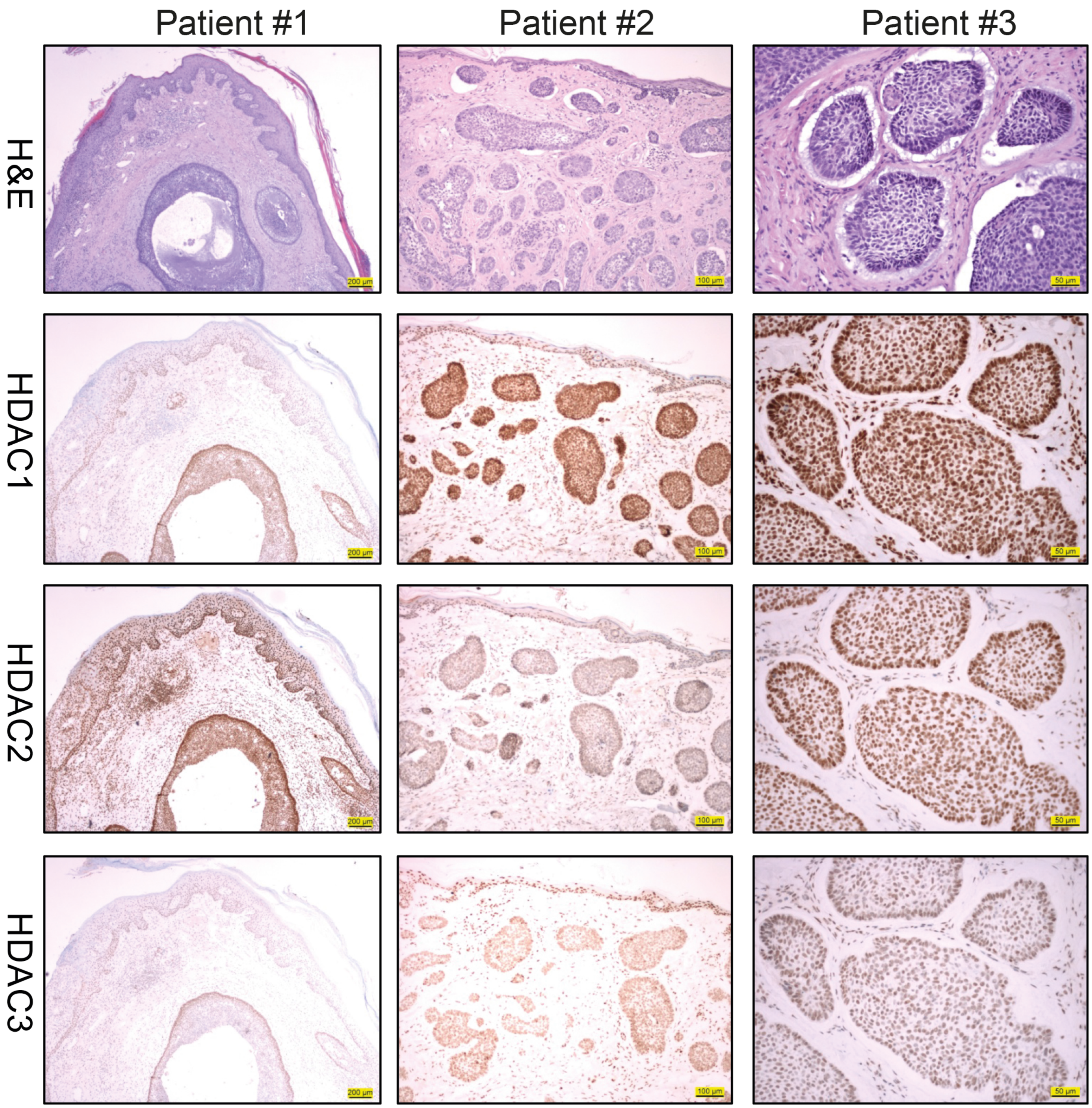

negative control: secondary antibody, only;

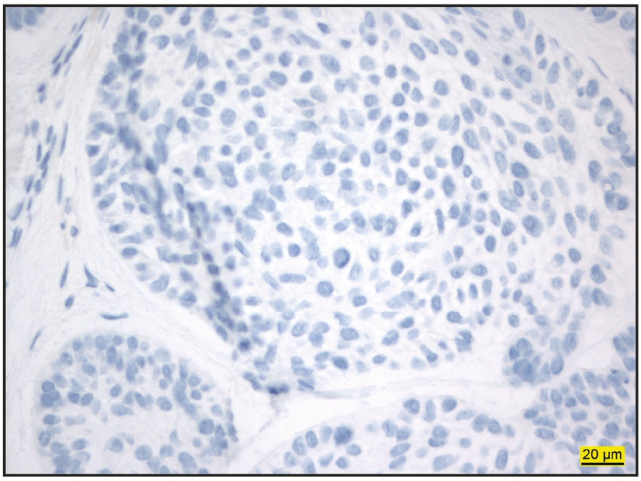

Gruber et al., Suppl. Figure 8

Supplementary figure legends:

Suppl. Figure 1: 4SC-202 does not affect primary ciliogenesis in HH-responsive Daoy cells

Confocal imaging of primary cilia of Daoy cells treated with 4SC-202 or solvent only (DMSO). To visualize and monitor the primary cilium, Daoy cells were stably transduced with a constitutively active form of GFP-tagged SMO (SmoM2-GFP) that localizes to and labels the primary cilium. Primary cilia of Daoy cells are indicated by arrows. DNA of Daoy cells were stained with DAPI. Z-stack images of GFP tagged SmoM2 and DAPI were acquired on a confocal microscope (Leica). Labeling of primary cilia was done as described previously by Gruber et al. [25].

Suppl. Figure 2: RNAi-mediated inhibition of class I HDAC expression and its effect on HH/GLI target gene expression.

(a-c) qPCR validation of siRNA mediated knock-down of HDAC1 (a), HDAC2 (b) and HDAC3 expression (c) in SAG or control treated Daoy cells. (d) GLI1 mRNA expression in SAG or control treated Daoy cells transfected with siRNA against HDAC1, HDAC2 or HDAC3. (e) HHIP mRNA expression in SAG or control treated Daoy cells transfected with siRNA against HDAC1, HDAC2 or HDAC3. SAG: Smoothened agonist;

Suppl. Figure 3: Validation of LSD1 knockdown in Daoy cells

qPCR analysis of LSD1 knockdown efficiency in Daoy cells transfected with control (siControl) or specific LSD1 siRNA (siLSD1). Transfection of siLSD1 reduced LSD1 mRNA levels by more than 90%. For the specific LSD1 knockdown the ON-TARGET plus siRNA pool was used, control cells were transfected with ON-TARGET plus non-targeting siRNA.

Suppl. Figure 4: The HDAC 1, 2 and 3 inhibitors 4SC-202 and entinostat reduce proliferation of SMOi-sensitive and SMOi-resistant Daoy cells

Subconfluent cells were treated with respective concentrations of 4SC-202 or entinostat for 90 hours and cell proliferation and viability were determined by incubation with Alamar blue. Left panel: SMOi sensitive, unmodified Daoy cells; right panel: SMOi resistant shSUFU Daoy cells; note that growth of SMOi sensitive wild-type as well as SMOi resistant shSUFU Daoy cells is affected by 4SC-202 treatment.

Suppl. Figure 5: The class-I HDACi 4SC-202 increases lysine27 acetylation of histone 3 at the PTCH promoter

ChIP analysis of histone acetylation of the PTCH promoter in response to HDAC inhibition by 4SC-202 treatment. Anti-H3K27acetylation and isotype control IgG antibodies were used for precipitation, respectively. Enrichment of histone-bound PTCH promoter DNA of control or 4SC-202 treated GLI1 expressing cells was measured by qPCR.

Suppl. Figure 6: 4SC-202 reduces GLI3 activity by shifting the Gli3 Activator (Gli3A) to Gli3 Repressor (Gli3R) ratio towards the Gli3R

(a) Western blot analysis of Med1 cells exposed to Vismodegib (1 µM), Hedgehog pathway inhibitor-1 (HPI-1 20 µM) as well as increasing concentrations of 4SC-202 (0.03 µM to 3 µM). 4SC-202 led to a decrease in full length Gli3A and at the same time an increase in Gli3R, thereby lowering the Gli3A:Gli3R ratio. (b) Densitometric quantification of the Gli3A:R ratio based on the Western blot analysis shown in (a).

Suppl. Figure 7: *ptch1/p53* deficient BCC cells express high levels of Hdacs 1, 2 and 3

(a) Western blot analysis of Hdac1/2/3 expression in *ptch1/p53* deficient murine BCC (mBCC) cells. Vismodegib (1 µM) did not affect expression levels of Hdac1/2/3. Total Erk 1/2 protein expression served as loading control.

Suppl. Figure 8: HDAC1/2/3 expression in human BCC

Immunohistochemistry analysis of HDAC1, HDAC2 and HDAC3 expression in BCC skin samples of three patients aged between 67 and 85 years. Magnification is 40x, 100x and 200x for patients #1, #2 and #3, respectively. Stainings without primary antibody were applied as negative controls. A representative negative control staining of BCC from patient #3 is shown.

Extended materials and methods

Cell culture and in vivo assays

For Hh/Gli reporter assays 2.5 x 10^4 NIH/3T3 Gli reporter cells (AMS Biotechnology) were seeded into 96 well plates and incubated overnight. One hour prior to stimulation with 1 µg/ml recombinant Shh (R&D Systems) cells were pretreated with respective compounds. The GLI-dependent activity of reporter luciferase was measured after 24h with the ONE-Glo™ Luciferase Assay System (Promega) and normalized to constitutively expressed renilla luciferase. The viability was assayed using the CellTiter-Fluor™ Kit (Promega).

For analyzing HH/GLI activity in the human Daoy cell line (ATCC HTB-186), cells were kept confluent for at least 48h and starved in 0.5% FBS (Sigma-Aldrich) overnight prior to stimulation with 100 nM SAG. Chemicals or control solvents were added 2h prior to SAG stimulation. Daoy cells were cultured in MEM (Sigma-Aldrich) supplemented with 10% FBS (Sigma-Aldrich) and antibiotics (Penicillin-Streptomycin, Sigma-Aldrich). The following chemicals were used: Smoothened agonist SAG (Axxora), vismodegib and entinostat (LC Laboratories), OG-L002, SAHA/vorinostat and 4SC-202 (4SC AG). The inhibitory activity of all compounds was validated in target-specific test assays.

A PanQinase® activity assay including 283 kinases was performed to exclude unspecific activity of 4SC-202 on kinases (suppl. Table 2).

For *in vivo* experiments mice were kept in individually ventilated cages (IVC) under specific pathogen-free conditions (SPF). All animal experiments were performed in compliance with the national requirements. The allograft assay was performed by injecting 10^6 BSZ2 cells [1] subcutaneously into the flanks of NOD-scid IL2Rgamma^{null} (NSG) mice with 25% Matrigel (BD Biosciences). Mice were randomized into 2 groups and treated with 4SC-202 (80 mg/kg/day per oral gavage) or solvent (2% hydroxypropylmethylcellulose derivative (HPMC, Methocel E19) solution from Dow Wolff Cellulosics GmbH) when tumors reached 400 mm^3. Mice were sacrificed after 7 days of treatment. Tumor volume was measured every 2-3 days with a caliper and calculated according to the formula V=4/3*π*length/2*width/2*height/2.

For AlamarBlue assays, cells were seeded at 2% confluency in 96-well plates and 24h later treated with the respective compounds as indicated in the text. Cell viability was determined by adding 10 µL alamarBlue solution (AbD Serotec) to each well, followed by measuring fluorescence (excitation 560 nm/emission 590 nm) 6h afterwards. The percentage of viable cells was normalized to the amount of viable cells in the respective control

RNA isolation and quantitative PCR (qPCR)

Total RNA was isolated using TRI reagent (Sigma-Aldrich) according to manufacturer's protocol followed by LiCl precipitation. 2 µg of total RNA was used for cDNA synthesis using M-MLV reverse transcriptase (Promega). qPCR was done on a Rotor Q (Qiagen) using GoTaq 2x qPCR Mastermix (Promega). qPCR primers are listed in suppl. Table 1.

Western blot analysis, Chromatin Immunoprecipitation (ChIP) and immunohistochemistry

For protein detection by Western blot analysis the following primary antibodies were applied: anti-GLI1 (V812), anti-Beta Actin (D6A8), anti-β-Tubulin (9F3, all Cell Signaling) and anti-SUFU (sc-10933, Santa Cruz Biotechnology). For the analysis of Gli3 processing, polyclonal anti-Gli3 antibody (AF3690, R&D Systems) was used. Primary antibodies were detected using horseradish peroxidase (HRP)-conjugated secondary antibodies (Cell Signaling). ChIP assays were conducted using the SimpleChIP Kit (Cell Signaling) with cross-linked chromatin immunoprecipitated overnight with either anti-H3K27ac antibody or anti-MYC-tag antibody (9B11, Cell Signaling) or mouse IgG isotype control. Precipitated chromatin was quantified by qPCR with PTCH1 specific primers [2]. Immunohistochemistry was done on FFPE tissue of three different skin specimens with diagnosis of BCC. In brief, 4 µm sections were stained using the following primary abs: anti-HDAC-1 (rabbit, ab19845, Abcam (Cambridge, UK), 1:2000), anti-HDAC-2 (rabbit, ab16032, Abcam (Cambridge, UK), 1:250) and anti-HDAC-3 (rabbit, sc-11417, Santa Cruz Biotechnology (Dallas, Texas), 1:100) with pH9 antigen retrieval buffer (Dako, Glostrup, Denmark) for all antibodies. The immunohistochemical stainings were performed on a Benchmark Ultra platform with the OptiView DAB IHC detection kit (Ventana (Arizona, USA)).

RNA interference

SUFU knockdown was done by lentiviral shRNA transduction as described in [3]. The SUFU targeting shRNA construct was selected from the Mission TRC shRNA library (TRCN0000019466, Sigma) and a scrambled shRNA served as control (SHC002, Sigma). LSD1 was knocked down using the ON-TARGET plus Human KDM1A siRNA (L-009223-00-0005, Dharmacon) according to manufacturer's instructions. The ON-TARGET plus Non-targeting siRNA served as control (D-001810-01-05, Dharmacon).

Statistical analysis

Graph design and statistical analysis was done using Graphpad Prism (GraphPad Software). Error bars represent the +/- standard error of the mean (SEM) of at least two experiments.

References:

1. So PL, Langston AW, Daniallinia N, Hebert JL, Fujimoto MA, Khaimskiy Y, Aszterbaum M, Epstein EH, Jr. Long-term establishment, characterization and manipulation of cell lines from mouse basal cell carcinoma tumors. *Exp Dermatol* 2006;**15**: 742-50.

2. Winklmayr M, Schmid C, Laner-Plamberger S, Kaser A, Aberger F, Eichberger T, Frischauf AM. Non-consensus GLI binding sites in Hedgehog target gene regulation. *BMC Mol Biol* 2010;**11**: 2.

3. Kasper M, Regl G, Eichberger T, Frischauf AM, Aberger F. Efficient manipulation of Hedgehog/GLI signaling using retroviral expression systems. *Methods Mol Biol* 2007;**397**: 67-78.
